# Effects of exogenous and endogenous cues on attentional orienting in deaf adults

**DOI:** 10.3389/fpsyg.2022.1038468

**Published:** 2022-10-06

**Authors:** Yunsong Li, Meili Luo, Xilin Zhang, Suiping Wang

**Affiliations:** ^1^Philosophy and Social Science Laboratory of Reading and Development in Children and Adolescents (South China Normal University), Ministry of Education, Guangzhou, Guangdong, China; ^2^Guangdong Provincial Key Laboratory of Mental Health and Cognitive Science, South China Normal University, Guangzhou, Guangdong, China; ^3^School of Psychology, South China Normal University, Guangzhou, Guangdong, China

**Keywords:** deaf adults, endogenous attention, exogenous attention, cue-target paradigm, attentional orienting

## Abstract

Adults who are deaf have been shown to have better visual attentional orienting than those with typical hearing, especially when the target is located in the periphery of the visual field. However, most studies in this population have assessed exogenous visual attention orienting (bottom-up processing of external cues) rather than endogenous visual attention orienting (top-down processing of internal cues). We used a target detection task to assess both types of visual attention orienting. A modified cue-target paradigm was adopted to assess the facilitation effects of exogenous and endogenous cues during short and long inter-stimulus intervals (ISI), using a 2 (Group: deaf/typically hearing) * 2 (Location: central/peripheral) * 2 (Cue Type: exogenous/endogenous) mixed factorial design. ANOVAs showed that both exogenous cues and endogenous cues can facilitate deaf adults’ visual attentional orienting, and the facilitation effect of exogenous cues on attention orienting was significantly stronger for deaf participants than hearing participants. When the ISI was long, the effect was significantly stronger when the exogenous cue appeared in the periphery of the visual field. In the periphery, deaf adults benefited most from exogenous cues, whereas hearing adults benefited most from endogenous cues. The results suggest that not only exogenous cues but also endogenous cues can facilitate deaf adults’ visual attentional orienting. However, the effect of exogenous cues appears to be greater, especially when the stimulus appears in the peripheral visual field.

## Introduction

In daily life, people integrate information from different sensory channels (visual, auditory, tactile, etc.) into a unified, coherent, and meaningful perception. When one sensory channel does not or cannot provide information, the other sensory channels become more perceptive, a phenomenon known as sensory compensation. For example, people who are deaf show an advantage in visual attention that compensates for their auditory disadvantage (Rettenbach et al., 1999; Buckley, et al., 2010; Pavani and Bottari, 2012; Alencar et al., 2019). This advantage is especially evident when the stimulus appears in the peripheral location of the visual field (Loke and Song, 1991; Bavelier et al., 2000, 2001; Bosworth and Dobkins, 2002; Sladen et al., 2005). This compensation may be temporal, evident in the shortening of reaction time in response to peripheral stimuli (Pavani and Bottari, 2012), or it may be spatial, evident in sensitivity to objects in a wider field of vision (Buckley, et al., 2010). For example, adults who are deaf show better detection ability to objects far from the central visual field, ranging from parafoveal to marginal visual fields, including 3° (Chen et al., 2006; Bottari et al., 2010), 20° (Colmenero et al., 2004), and larger visual angles (Codina et al., 2011).

Deaf adults’ advantage in visual attention at the periphery has been demonstrated in studies using a range of methodologies. For example, Bottari et al. compared the visual attention processing of a hearing group and a deaf group using a simple detection task and a shape discrimination task. The deaf group responded faster than the hearing group on a simple detection task, regardless of whether the stimulus appeared in the center or the periphery of the visual field. Moreover, the hearing group responded more slowly in judging peripheral location stimulus than that in judging central location, but the deaf group completed the task equally well at both locations (Bottari et al., 2010). Prasad et al. also investigated attentional orientation to cues from different locations in the visual field, using saccade data and behavioral response data. The deaf participants had enhanced visual attention compared to the hearing participants, and the higher facilitation was found at the periphery in both groups (Prasad et al., 2015). Neville and Lawson asked deaf and typically hearing people to judge the direction of motion of randomly presented stimuli. When the stimulus appeared in the periphery of the visual field, the deaf group showed increases in attention-related potentials, with the magnitude being several times greater than what was recorded in the typically hearing group. However, there was no significant difference between the two groups in response to centrally located stimuli (Neville and Lawson, 1987).

A limitation of these literature is that most studies have compared deaf and hearing people on only one form of visual attention. Posner (1980) described attention as endogenous attention which is elicited by endogenous cues and exogenous attention which is elicited by exogenous cues. They represent the top-down and bottom-up processing of attention, respectively (Chica et al., 2013; Tang et al., 2016). Most studies have assessed visual attention in terms of exogenous attention. Although several studies have measured the endogenous visual attention of people who are deaf (Belardinelli et al., 2007; Bottari et al., 2008; Heimler et al., 2015; Prasad et al., 2022), the focus of these studies was whether deaf participants could use top-down strategies to suppress the influence of irrelevant exogenous stimuli to improve performance on a search task, rather than assess the independent influence of exogenous cue on deaf adults’ attention orient.

There is also an important inconsistency in the results of two studies on the visual attention of deaf and hearing participants. Heimler et al. (2015) recorded the eye movements of deaf and hearing participants while they searched for the location of a known stimulus target. Although the deaf participants had slower search speed than the hearing participants, they were less susceptible to the influence of exogenous stimuli in the goal-driven condition. However, Prasad et al. (2022) found no difference between deaf and hearing participants in the ability to suppress spatial distractors that were unrelated to the target. One problem is that these two studies tested the ability of deaf participants to suppress the influence of unrelated exogenous stimuli when using endogenous visual attention, but they did not assess the attentional orienting elicited by endogenous cues.

When only endogenous cues play a role, it is unknown whether there is a difference in attentional orienting between adults who are deaf and those who have typical hearing. If there is a difference, it is unclear whether it would be similar to the differences that have been observed in attentional orienting elicited by exogenous cues. Therefore, we studied whether deaf participants showed enhanced visual attention to both endogenous cues and exogenous cues, relative to hearing participants. The same task was used to assess the effects of both types of cues. This design allowed us to investigate whether the deaf and hearing participants differed in their visual attention processing, specifically whether they relied more on bottom-up or top-down processing. The results will lead to a better understanding of deaf and hearing participants’ visual attention processing.

We used a cue-target paradigm to investigate participants’ visual attentional orienting. This is a classic paradigm for investigating spatial attention (Posner and Cohen, 1984). Posner and Cohen (1984) presented a visual cue on the left or right peripheral location of a display; after a period of time the target appeared in either the same peripheral location of the cue or in the middle of the display. Participants were asked to press a key on a keyboard as soon as they perceived the target. A facilitation effect is said to occur when participants respond faster to a target presented at a cued location (the cue and target are at the same location) than at an uncued location (the cue and target are at opposite locations). We improved this paradigm by adding endogenous cue conditions, and by including stimulus location as a factor in the design. In addition, previous studies have found that exogenous attention and endogenous attention have different time courses. Endogenous cues come into play later than exogenous cues (Muller and Rabbitt, 1989; Nakayama and Mackeben, 1989; Keefe and Störmer, 2021). Thus, in this experiment, we use long and short inter-stimulus intervals (ISI) to investigate the effect of cues. The ISI of endogenous cues was 50 ms longer than that of exogenous cues in both short and long ISI conditions.

## Materials and methods

### Participants

Forty-two adults who are deaf participated in this experiment, including 25 males. The average age of the participants was 34.51 ± 6.07 years old. All of them had the onset of hearing impairment before 2 years old, and all of them had the first-level hearing disability (the average hearing loss in both ears was >90 dB HL). We assessed participants’ familiarity with Chinese sign language using a 5-point scale (1: familiar; 5: unfamiliar). Thirty-seven of the 42 deaf participants were familiar with the sign language (score 3 or less). At the same time, 36 participants with normal hearing were recruited, including 20 males. The average age of the participants was 38.38 ± 6.65 years old. All the participants had normal vision and were right-handed. They were paid at the end of the experiment. The study was approved by the ‘The Ethics Committee of School of Psychology at South China Normal University.’ All participants gave written informed consent in accordance with the Declaration of Helsinki.

### Materials

The stimulus was presented on a 14-inch LCD display with a screen resolution of 1920 × 1080 pixels and a refresh rate of 60 Hz. The experimental program was programmed by E-Prime (Psychology Software Tools, Pittsburgh, PA, USA; version 2.0), and all visual stimuli were presented on a gray background. The participants were seated 60 cm away from the computer screen. Each participant completed the experiment alone in the laboratory.

**Figure 1:**
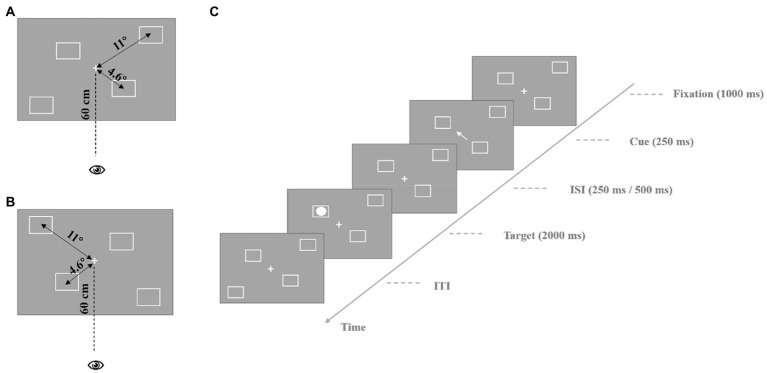
Process of endogenous cue experiment. **(A)** The stimulus parameters are presented in the experiment. The central location stimulus is presented at the participant’s 4.6° visual angle, the peripheral location stimulus is presented at the participant’s 11° visual angle, participant’s eyes are 60 cm away from the screen. **(B)** Different spatial distribution type of squares. The parameters of stimulus are the same as **(A)**. **(C)** The process of a single trail. ISI, inter-stimulus interval; ITI, inter-trial interval.

**Figure 2:**
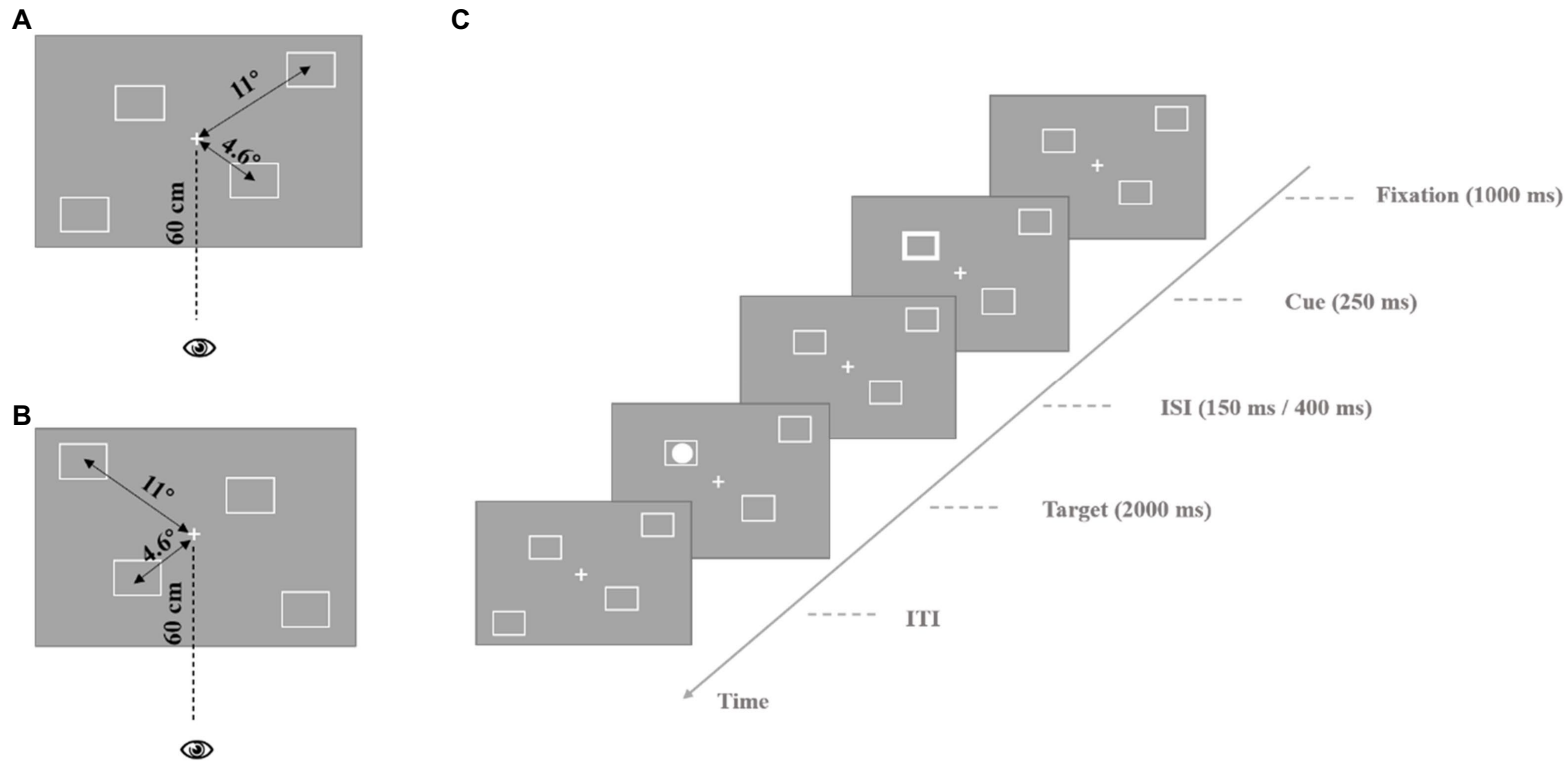
Process of exogenous cue experiment. **(A)** The stimulus parameters are presented in the experiment. The central location stimulus is presented at the participant’s 4.6° visual angle, the peripheral location stimulus is presented at the participant’s 11° visual angle, participant’s eyes are 60 cm away from the screen. **(B)** Different spatial distribution type of squares. The parameters of stimulus are the same as **(A)**. **(C)** The process of a single trail. ISI, inter-stimulus interval; ITI, inter-trial interval.

The experimental materials for the endogenous cues are shown in Figure 1. In the fixation screen, the center of the screen presents a white “+” as a central fixation, and there are two white squares on each side of the screen, one is 4.6° from the central fixation, and the other is 11° from the central fixation. For the four squares, we designed two different types of spatial distributions (see Figures 1A,B or 2A,B). Each participant was presented with only one type of these materials, and the materials were balanced between participants. On the cue display screen, the central fixation became an arrow pointing to the location of one of the squares to lead the participant’s endogenous attention, and then changed back to a white “+.” The target stimulus was a white solid circle inside the square, and the participants were asked to detect its position when it appeared. The exogenous cue stimulus materials were similar to the endogenous cue stimulus materials, except instead of an arrow indicating a direction on the screen, the square in different locations suddenly became thicker to trigger the participants’ exogenous attention.

### Procedure

The experiment adopted a mixed 2 (group: deaf and hearing) × 2 (location: central and peripheral) × 2 (cue type: endogenous and exogenous) design, the location and cue type are within-subject factors. All analyses were conducted twice, once after a short ISI and once after a long ISI. Each participant was required to complete both the endogenous cue experiment and the exogenous cue experiment. The order of the two experiments was counterbalanced between participants.

The process of a single trial in the endogenous cue experiment was as follows. First, the fixation was presented for 1,000 ms, and then, the central fixation turned into an arrow pointing to the location of one of the squares as a cue for 250 ms. After 250 ms or 500 ms, the target stimulus appeared for 200 ms at the same location (valid cue) or at the diagonal location (invalid cue) in the direction of the cue. The participants were asked to judge the location of the target. The different locations of the four squares from left to right were represented by the “2,” “4,” “6,” and “8” keys on the keyboard. Participants were asked to focus on the central fixation until the end of the experiment. The formal experiment included 128 trials (with a 3:1 ratio of valid and invalid cue trials). The experimental process is shown in Figure 1C.

The process of single trial of exogenous cue experiment: Firstly, the fixation was presented for 1,000 ms, and then the square at a certain location would become thicker for 250 ms. After 150 ms or 400 ms, the target stimulus appeared at the same location or diagonal location in the direction of the cue, lasting for 2,000 ms. When the target appeared, the participants were asked to judge the location of the target. The different locations of the four squares from left to right were represented by “2,” “4,” “6,” and “8” on the keyboard. Participants were asked to focus on the central fixation throughout the experiment until the end. The formal experiment included 128 trials (with a 1:1 ratio of valid and invalid cue trials). The experimental process is shown in Figure 2C.

## Results

Participant whose average accuracy was lower than 80% or reaction time was more than 1,000 ms, and whose individual reaction time or accuracy exceeded the total average reaction time and accuracy by plus or minus 3 standard deviations were eliminated. If the participants’ response time or accuracy exceeded this limit, all their data will be removed. The remaining 40 participants who are deaf and 34 hearing participants were included in the final analysis.

### Short ISI condition

#### Accuracy

All the participants were able to complete the task well, the average accuracy in each condition was more than 95%. There is a ceiling effect. Therefore, there is no further discussion of accuracy.

#### Reaction time

In all conditions, it was found that the reaction time of the participants to the cued location was significantly shorter than uncued location by paired *t*-test (*p* < 0.01). Only when exogenous cue was presented in the central location of hearing participants, the facilitation effect was marginally significant, *t*(33) = 1.90, *p* = 0.066. Therefore, the size of the cueing effect was taken as the dependent variable of this experiment, that is, in the same condition, the reaction time of the participant to the uncued location (cue and target appear at opposite locations) minus the reaction time of the cued location (cue and target appear at the same location) was represented. The descriptive statistics of cueing effect in different conditions are shown in Table 1.

**Table 1:** Mean cueing effect (ms) and standard error of deaf and hearing groups on stimuli from different cue types and locations when there is a short ISI.

	Deaf adults		Hearing control	
	Endogenous cue	Exogenous cue	Endogenous cue	Exogenous cue
Central	50.36 (11.41)	57.1 (8.04)	65.06 (14.85)	25.91 (13.62)
Peripheral	114.33 (10.55)	137.52 (7.15)	148.53 (17.86)	115.42 (13.93)

**Figure 3:**
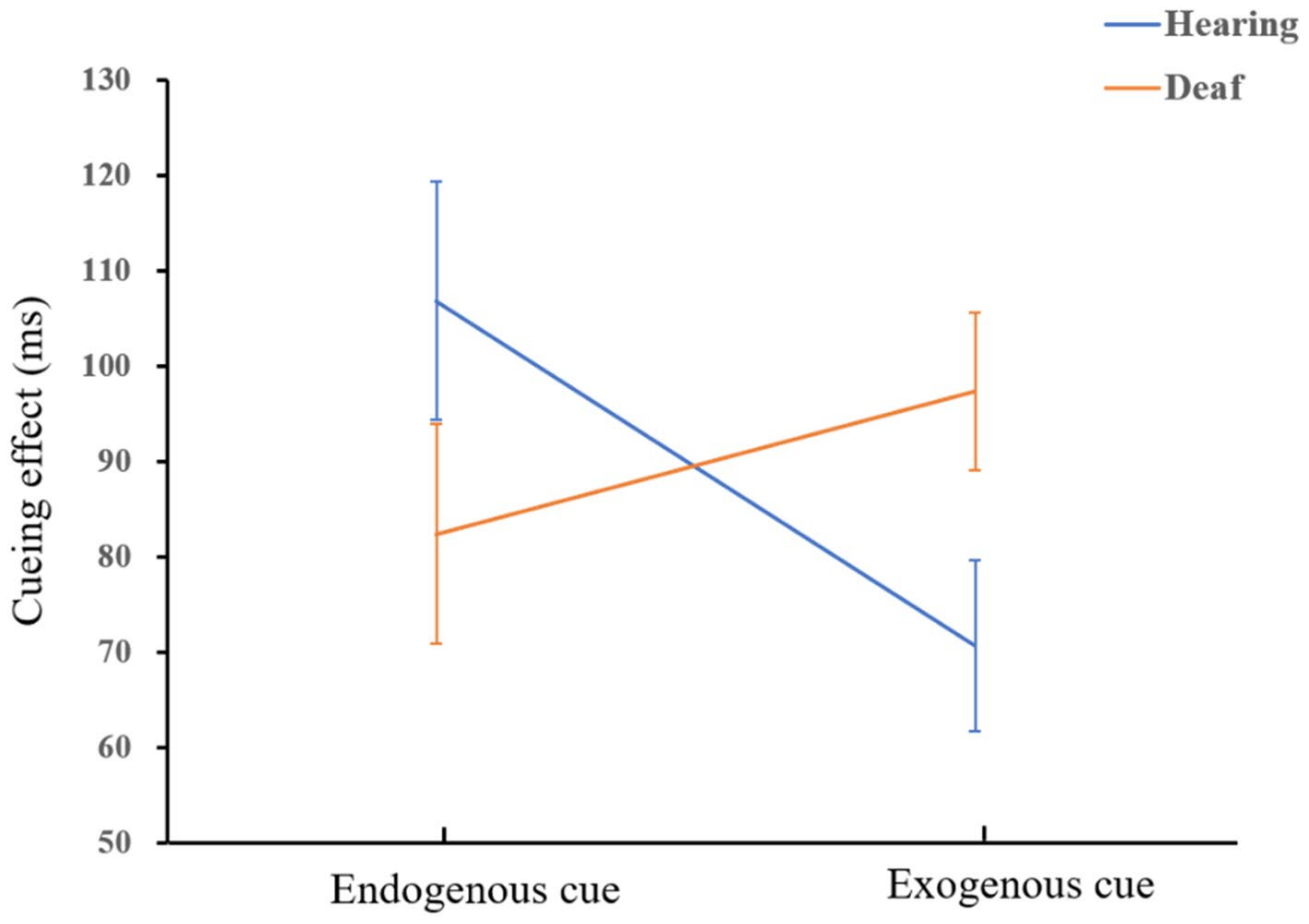
The interaction of group and cue type when there is a short ISI.

We conducted a 2 × 2 × 2 mixed ANOVA with the group (deaf and hearing), cue type (endogenous and exogenous), and location (central and peripheral) as independent variables, and cueing effect as a dependent variable. The results showed that the main effect of location was significant, *F*(1,72) = 135.64, *p* < 0.001, $\eta_{p}^{2}$ = 0.65. Participants responded significantly faster to the peripheral location than to the central location. The interaction of cue type and group was significant, *F*(1,72) = 4.49, *p* = 0.04, $\eta_{p}^{2}$ = 0.06. Simple effect analysis showed that exogenous cues had a greater facilitation effect on people who are deaf than hearing people, *F*(1,72) = 4.81, *p* = 0.03, $\eta_{p}^{2}$ = 0.06. There was no significant difference in the facilitation effect of endogenous cues between hearing control group and deaf adult group, *p* = 0.10. A diagram of the interaction is shown in Figure 3.

### Long ISI condition

Similarly, we found that reaction time of the participants to the cued location was significantly shorter than uncued location in all conditions by paired *t*-test (*p* < 0.01). Therefore, we took the size of the cueing effect as our dependent variable, namely, the reaction time of the participants to the uncued location minus the reaction time of the cued location in the same condition. The descriptive statistics of cueing effect in different conditions are shown in Table 2.

**Table 2:** Mean cueing effect (ms) and standard error of deaf and hearing groups on stimuli from different cue types and locations when there is a long ISI.

	Deaf adults		Hearing control	
	Endogenous cue	Exogenous cue	Endogenous cue	Exogenous cue
Central	49.51(13.76)	78.45 (6.23)	50.04 (17.58)	40.62 (13.26)
Peripheral	100.76(11.18)	140.37 (8.18)	139.87 (16.8)	92.94 (13.89)

**Figure 4:**
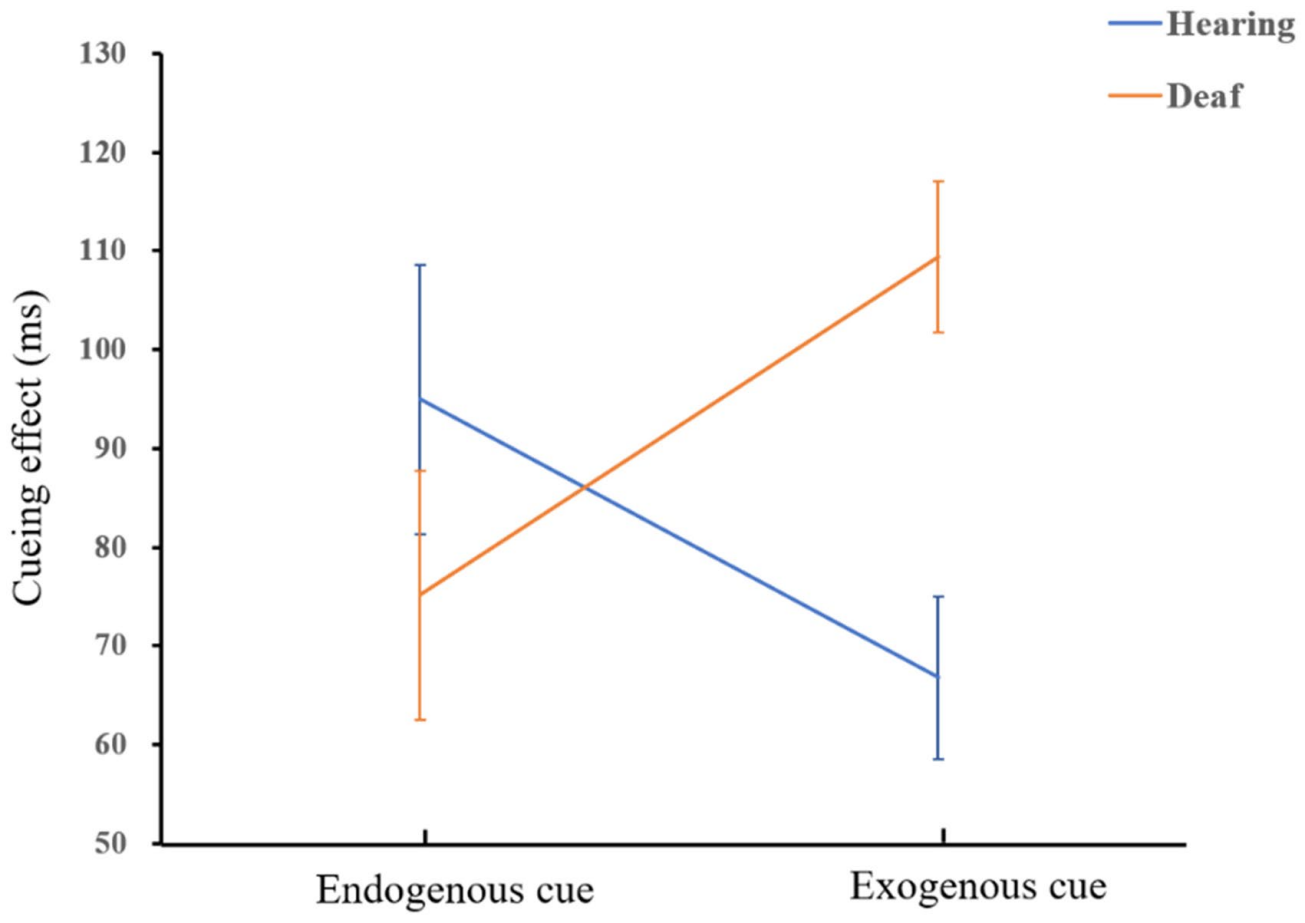
The interaction of group and cue type when there is a long ISI.

The results of mixed ANOVA showed that the main effect of the location was significant, *F*(1,72) = 80.2, *p* < 0.001, $\eta_{p}^{2}$ = 0.53. Participants responded significantly faster to the peripheral location than to the central location. The interaction of cue type and group was significant, *F*(1,72) = 7.4, *p* = 0.008, $\eta_{p}^{2}$ = 0.09. Simple effect analysis showed that exogenous cues had greater facilitation effect on the deaf adults group than hearing control group, *F*(1,72) = 14.34, *p* < 0.001, $\eta_{p}^{2}$ = 0.17. There was no significant facilitation effect of endogenous cues between deaf and hearing groups, *p* = 0.29. A diagram of the interaction is shown in Figure 4.

**Figure 5:**
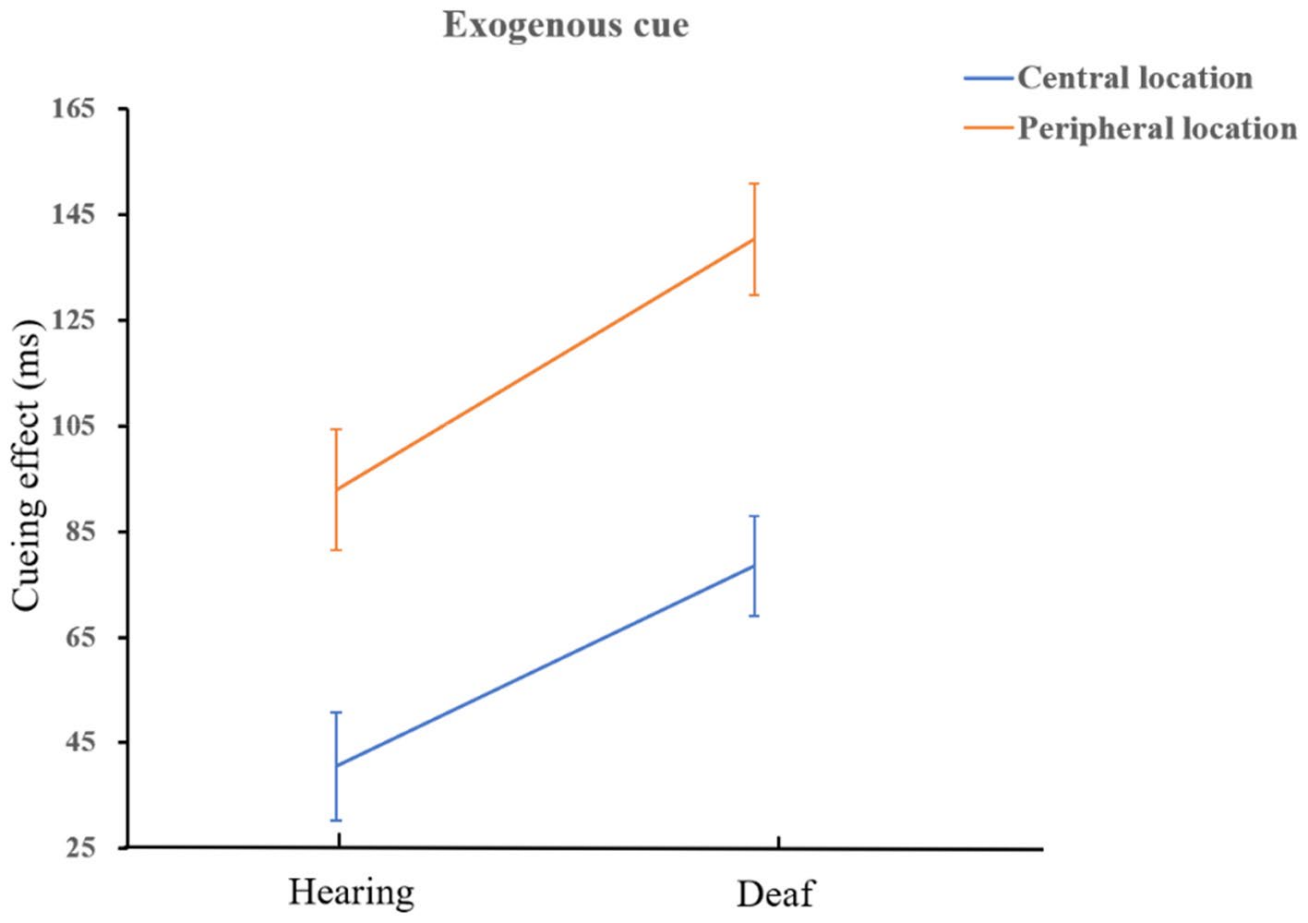
The interaction of group and location when cues are exogenous.

**Figure 6:**
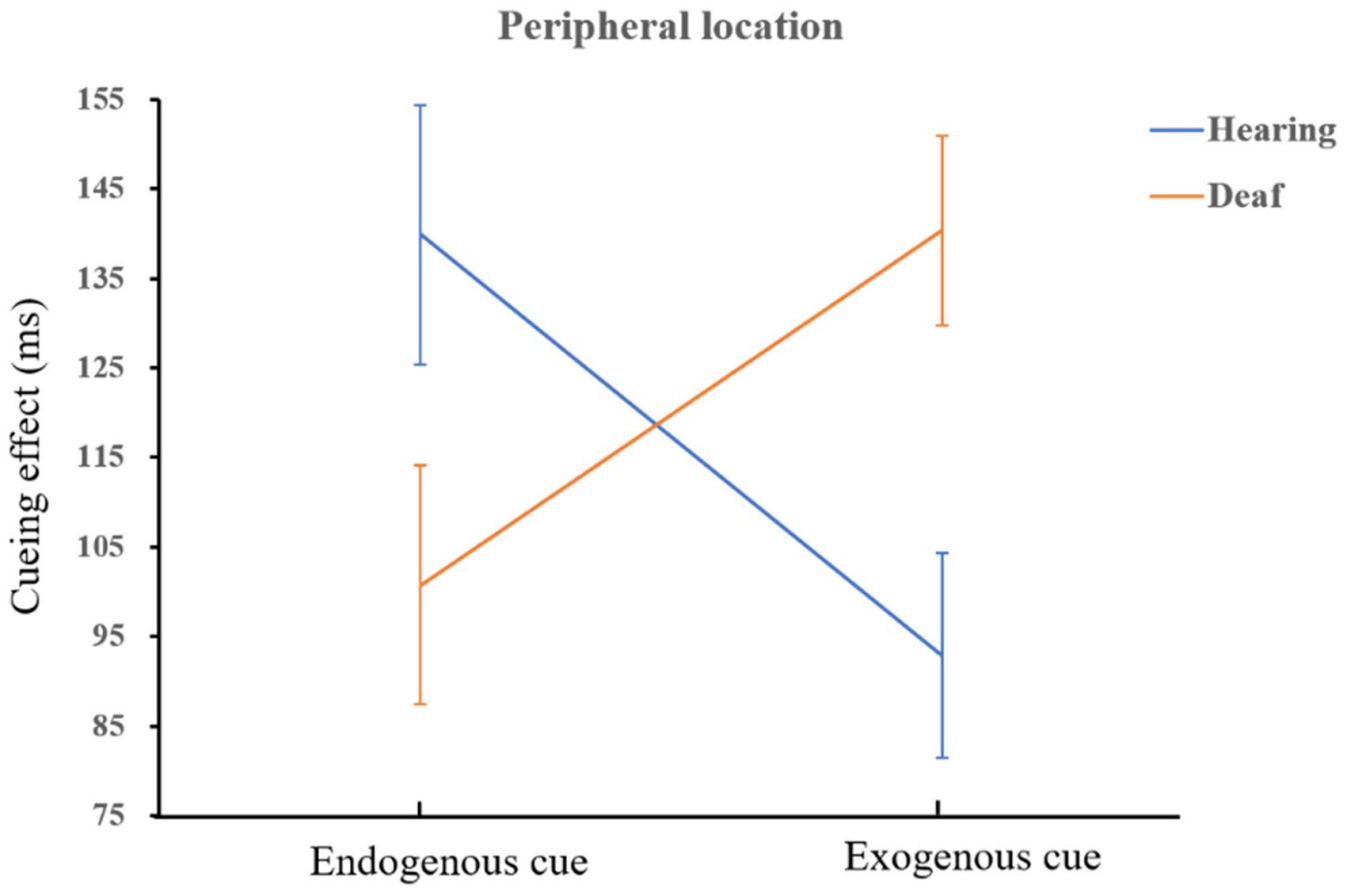
The interaction of group and cue type when stimulus is presented in peripheral location.

The interaction of cue type, location, and group was marginally significant, *F*(1,72) = 3.63, *p* = 0.06, $\eta_{p}^{2}$ = 0.05. Simple effect analysis showed that in the central location, the facilitation effect of exogenous cues on deaf was greater than that of hearing participants, *F*(1,72) = 7.35, *p* = 0.008, $\eta_{p}^{2}$ = 0.09; Similarly, in peripheral location, the facilitation effect of exogenous cues on deaf was greater than that of hearing participants, *F*(1,72) = 9.27, *p* = 0.003, $\eta_{p}^{2}$ = 0.11. It shows that people who are deaf are more sensitive to exogenous cues than hearing, and it is very easy to capture bottom-up information. A schematic diagram of the interaction is shown in Figure 5. Both deaf and hearing groups showed a more endogenous cueing effect in peripheral location than in central location which is in line with the main effect of location (normal hearing group: *F*(1,72) = 41.67, *p* < 0.001, $\eta_{p}^{2}$ = 0.37; deaf group: *F*(1,72) = 15.95, *p* < 0.001, $\eta_{p}^{2}$ = 0.18). We also found that when the cue indicated the peripheral location, the facilitation effect of endogenous cues on hearing group was better than that of exogenous cues, *F*(1,72) = 6.07, *p* = 0.02, $\eta_{p}^{2}$ = 0.08; on the contrary, exogenous cues had a better effect on people who are deaf than that of endogenous cues, *F*(1,72) = 5.08, *p* = 0.03, $\eta_{p}^{2}$ = 0.07. The schematic diagram is shown in Figure 6.

## Discussion

The results supported the hypotheses (1) Both exogenous and endogenous cues had a significant facilitation effect on the visual attention of deaf participants. Participants were significantly faster to detect the targets at a cued location than an uncued location. (2) When exogenous and endogenous cues were assessed by the same task, during both short ISI and long ISI, the facilitation effect of exogenous cues on visual attention was stronger for deaf participants than hearing participants, whereas the facilitation effect of endogenous cues was not significantly different between the two groups. (3) When the interval between cue and target was longer, the two types of cues in the peripheral location but not the central location had opposite facilitation effects on deaf and hearing participants’ visual attention. For the participants who were deaf, exogenous cues had a stronger effect than endogenous cues; the reverse was true for hearing participants. The results suggest that the visual attention of people who are deaf is more easily attracted by the bottom-up spatial stimulus, and it is relatively difficult to shift attention to a certain spatial location in a top-down manner. In contrast, people with normal hearing are more likely to be prompted by endogenous cues in the peripheral visual field, indicating that they tend to use a top-down manner to shift their attention to the peripheral visual field.

The deaf participants in our sample responded better to the target at the peripheral location than to the central location when using endogenous attention to complete detection task, and this result differed from the results of other studies. Bottari et al. (2008) found that after excluding the influence of exogenous cues, only profoundly deaf participants perceived changes in the visual field during the distributed attention task. Bottari’s results also showed that when only endogenous attention was used, these participants were more sensitive to the changes of objects in the central location than in the peripheral location (Bottari et al., 2008). The inconsistent results may be due to differences in experimental paradigms. In Bottari et al. (2008) distributed attention task, participants were asked to scatter their attention to various locations on the screen. The requirements of this task are relatively vague, and the ratio of the participants’ attention to each location of the screen (central/peripheral) cannot be determined. Because deaf participants’ eye movement trajectories were not recorded, it cannot be determined whether people who are deaf tend to focus their attention more on the central location.

In our experiment, arrows were used to indicate the orientation, and this endogenous cue was more directional, prompting the participants to shift their attention to a certain location on the screen. This task can better arouse the participants’ endogenous attention than the research task. However, our experiment did not eliminate or control the attraction of the exogenous stimulus when the target was present, as Bottari’s task did. There is a possibility that the target triggers the participants’ exogenous attention, and the facilitation effect of the exogenous stimulus is stronger in the peripheral than in the central location. The overall facilitation effect reflects the joint effects of endogenous attention and exogenous attention.

What are the differences in the facilitation effects of the two different types of cues on visual attention? The results of this experiment show that people who are deaf can make better use of exogenous cues, but make poor use of endogenous cues compared with the hearing group in the peripheral location. In the past, this question was discussed based on data from hearing participants. Geweke, Pokta, and Störmer investigated participants’ spatial attention at central and peripheral locations when the cues were endogenous and exogenous. The results showed that participants’ reaction time was significantly longer for endogenous cues than exogenous cues, and it was more time-consuming for participants to move their attention to a distant location regardless of the type of cue (Geweke et al., 2021). Guzman-Martinez’s experiment also found a difference between the two types of attention in promoting individual target search performance. Endogenous attention had advantages in processing multiple targets, while exogenous attention had advantages in processing a single target (Guzman-Martinez et al., 2011). This study provides additional evidence from deaf individuals regarding the difference between the two types of visual attention.

At the same time, we noticed that the two types of cues in peripheral location produced opposite facilitation patterns for the deaf and hearing groups. Namely, the facilitation effect of exogenous cues on deaf participants was greater than that of endogenous cues, while the facilitation effect of endogenous cues on hearing participants was greater than that of exogenous cues. One possible reason is that people tend to inhibit peripheral visual field stimulation in order to better focus attention around the fovea. Thus, individuals show inhibition of exogenous cues in peripheral locations. Only when the task or top-down information requires the individual to focus on the location that should be suppressed, the individual uses the executive control system to transfer the attention to the peripheral location without inhibition. Bao and Poppel’s study, for example, found greater inhibition of return (in some ISI, hearing participants were significantly slower to respond to cued locations than to uncued locations) when a target appeared farther from the fovea (Bao and Poppel, 2007). Yang, Zhang, and Bao also found that exogenous cues produced inhibition of return in perifoveal location in hearing participants, whereas endogenous cues produced the facilitation effect (Yang et al., 2015). According to the results of our experiment, adults who are deaf are more likely to be facilitated, rather than inhibited by, bottom-up stimuli located in the peripheral visual field.

For the deaf adults, it was more difficult to use endogenous cues to complete the task than exogenous cues. One possible reason is that due to their poorer executive control ability, it is difficult for them to use their executive control system to shift attention to further locations in the visual field in a shorter period of time. The study by Matthew et al. (2007) found that compared with normal hearing group, deaf group experienced a greater flanker interference effect in the peripheral visual field. This indicates that the people who are deaf have a weaker inhibitory control ability in the peripheral visual field. Similarly, Holmer et al. (2020) found that deaf participants with gaming experience had better attentional control than deaf group without gaming experience, arguing that this was due to the better anti-interference ability of deaf people who played games. Therefore, weaker executive control may be the reason why the deaf adults cannot make full use of endogenous cues.

Endogenous and exogenous cues can influence attentional orienting, but their joint effects may also be important to study. In the past, this issue was considered in samples of hearing participants. One study examined the combined effect of the two types of cues presented sequentially (endogenous cues followed by exogenous cues) on participants’ attentional orienting. When the endogenous cue was effective, the exogenous cue also had a facilitation effect on target detection, whereas when the endogenous cue was ineffective, the exogenous cue had an inhibitory effect on the participants’ responses (Chen et al., 2012). Hopfinger and West also presented endogenous and exogenous cues in sequence, and found that the two types of cues differed in the duration of action and were activated at different electrode locations (Hopfinger and West, 2006). However, little is known about the combined effects of different types of attention in people who are deaf. A small number of studies have focused on this issue, but have not reached a unified conclusion (Heimler et al., 2015; Prasad et al., 2022). Further studies are needed to identify different paradigms than can provide stronger evidence of the combined effects on deaf adults.

The experimental results of this study can also provide some insights into special education. For example, this study found that exogenous cues elicited greater attentional orientations in deaf group than in hearing group, especially at peripheral visual field locations. This suggests that hearing impaired people are more likely to be disturbed by irrelevant stimuli in classroom learning or in jobs that require high concentration. Perhaps remedial training for this disadvantage is important.

There are also some deficiencies in our experiment. Due to the lack of eye movement data, we were unable to investigate the time spent in each stage of deaf participants’ attention shift after receiving endogenous cues. Nor can we judge whether the participants’ attention was focused on a specific location until the target appeared (as requested). These issues are open to further investigation.

## Conclusion

Both exogenous and endogenous cues can facilitate the visual attention orientation of people who are deaf. Exogenous cues have a stronger facilitation effect than endogenous cues, especially when stimuli appear in the deaf adult’s peripheral visual field.

## Data availability statement

The raw data supporting the conclusions of this article will be made available by the authors, without undue reservation.

## Ethics statement

The studies involving human participants were reviewed and approved by the Ethics Committee of School of Psychology at South China Normal University. The patients/participants provided their written informed consent to participate in this study.

## Author contributions

SW and XZ designed the research and reviewed the manuscript. YL and ML performed the research and analyzed the data. YL and SW wrote the manuscript text. All authors contributed to the article and approved the submitted version.

## Funding

This work was supported by the National Natural Science Foundation of China (No. 32171051). The funders had no role in study design, data collection and analysis, decision to publish, or preparation of the manuscript.

## Conflict of interest

The authors declare that the research was conducted in the absence of any commercial or financial relationships that could be construed as a potential conflict of interest.

## Publisher’s note

All claims expressed in this article are solely those of the authors and do not necessarily represent those of their affiliated organizations, or those of the publisher, the editors and the reviewers. Any product that may be evaluated in this article, or claim that may be made by its manufacturer, is not guaranteed or endorsed by the publisher.
